# Coinfection of Human Herpesviruses 6A (HHV-6A) and HHV-6B as Demonstrated by Novel Digital Droplet PCR Assay

**DOI:** 10.1371/journal.pone.0092328

**Published:** 2014-03-24

**Authors:** Emily C. Leibovitch, Giovanna S. Brunetto, Breanna Caruso, Kaylan Fenton, Joan Ohayon, Daniel S. Reich, Steven Jacobson

**Affiliations:** 1 Neuroimmunology Branch, National Institute of Neurological Disorders and Stroke, National Institutes of Health, Bethesda, Maryland, United States of America; 2 Institute for Biomedical Sciences, School of Medicine and Health Sciences of The George Washington University, Washington, DC, United States of America; University of Utah, United States of America

## Abstract

The human herpesviruses HHV-6A and HHV-6B have been associated with various neurologic disorders partly due to the detection of elevated viral DNA levels in patients compared to controls. However the reported frequency of these viruses varies widely, likely reflecting differences in PCR methodologies used for detection. Digital droplet PCR (ddPCR) is a third generation PCR technology that enables the absolute quantification of target DNA molecules. Mounting evidence of the biological differences between HHV-6A and HHV-6B has led to their recent reclassification as separate species. As it is now especially relevant to investigate each virus, our objectives were to first design a multiplex HHV-6A and HHV-6B ddPCR assay and then to investigate the incidence of HHV-6A and HHV-6B coinfection in samples from healthy donors and patients with MS, a disease in which HHV-6 is thought to play a role. In our assessment of healthy donors, we observed a heretofore-underappreciated high frequency of coinfection in PBMC and serum, and found that our assay precisely detects both HHV-6A and HHV-6B chromosomally integrated virus, which has important implications in clinical settings. Interestingly, upon comparing the saliva from MS patients and healthy donors, we detected a significantly elevated frequency of coinfection in MS saliva; increased detection of HHV-6A in MS patients is consistent with other studies suggesting that this viral species (thought to be more neurotropic than HHV-6B) is more prevalent among MS patients compared to healthy donors. As the biology and disease associations between these two viral species differ, identifying and quantifying both species of HHV-6 may provide clinically relevant information, as well as enhance our understanding of the roles of each in health and disease.

## Introduction

Human herpesvirus 6 (HHV-6) comprises two ubiquitous human beta-herpesviruses: HHV-6A and HHV-6B. HHV-6A and HHV-6B share about 95% nucleotide identity, but some regions have up to 25% divergence [1], [2], which likely underlies the differences in tropism [3], drug susceptibilities [4] and disease associations between these two viruses. These differences have led to their recent reclassification as separate viral species [5].

Primary infection with HHV-6 occurs around the age of two, and as with other herpesviruses, latency persists for the life of the host. HHV-6B is the etiologic agent of roseola [6], a self-limiting febrile illness of early childhood. Primary infections with HHV-6 vary geographically; HHV-6B predominates in childhood infections in the US [6] and Japan [7], while a recent report suggests that HHV-6A may predominate in asymptomatic childhood infections in Zambia [8].

HHV-6A and HHV-6B have each been associated with diseases of the central nervous system (CNS) (reviewed in [9]) including multiple sclerosis (MS) [10], [11], encephalitis [12] and epilepsy (reviewed in [13]). These viruses are also known to reactivate following bone marrow [14], [15] and solid organ [16] transplant, causing complications that range from febrile illness [17] to fatal encephalitides [16], [18]. The association of the HHV-6 viruses with these disorders has been partly established by the detection of elevated viral DNA levels in patients compared with healthy controls. Many studies report PCR amplification of HHV-6A and HHV-6B from blood, saliva [19], [20] and urine [21], [22] of healthy adults. However, the reported frequencies of detection are wildly discrepant. For example in PBMC, the reported detection of HHV-6 in healthy adult donors ranges from 9% [23] to 90% [24], and in saliva from 9% [25] to over 80% [26]. These differences may be population-based, but they may also reflect the lack of a validated, standardized method for detecting HHV-6 viral DNA.

Since in most healthy individuals the concentration of HHV-6 in the peripheral blood is low, its detection is dependent on PCR sensitivity and subject to high inter-replicate variance. Nested PCR has proven to be a valuable tool for detecting low frequency events; however, it is not quantitative and is highly prone to false positive signals [27]. Though real time PCR is quantitative, its accuracy is limited by the need for an external calibrator [28]. A new, non-nested digital PCR technology has recently emerged that enables absolute quantification and offers an extraordinarily high degree of precision [28]. In digital PCR, DNA is distributed across multiple replicate reactions. These replicates enable the use of Poisson statistics for absolute quantification; the dynamic range increases proportionally with the number of replicates [28].

The method employed in this paper, digital droplet PCR (ddPCR) uses water-in-oil droplets to increase the number of replicates up to 20,000 nanoliter-sized droplets that each support PCR amplification. As the droplet volume is known, the fraction of positive droplets is used to calculate the absolute concentration of the target [28]. This system has recently been utilized to quantify bacteria [29] and viruses including HIV [30], [31] and CMV [32] in various sample types.

In light of increasing evidence regarding the biological differences between HHV-6A and HHV-6B, it is important that current research on this subject distinguish between the two. Therefore, we designed a multiplex ddPCR reaction for the simultaneous detection of HHV-6A and HHV-6B, with one primer set to amplify both viruses, and fluorescent probes that are specific for each. This SNP-like assay design enables comparable sensitivity and kinetics between amplification reactions, while differentiating HHV-6A and HHV-6B with high specificity and sensitivity. To our knowledge, this present study is the first report on the use of ddPCR for the detection of HHV-6, and the first report on its use for multiplexing two viruses. We report our findings on the quantitative detection of the HHV-6 viruses from human biological material including PBMC, serum and saliva. Notably, we demonstrate a high frequency of HHV-6A and HHV-6B coinfection in healthy controls, and this frequency is further elevated in the saliva of patients with MS, a disorder in which HHV-6 has been reported to play a role.

## Materials and Methods

### Ethics Statement

Prior to study inclusion, written informed consent was obtained from each subject (adult healthy volunteers and MS patients) in accordance with the Declaration of Helsinki. The study was reviewed and approved by the National Institutes of Health Institutional Review Board

### Clinical samples

The 59 MS patients were composed of 32 males and 27 females, 31 with relapsing-remitting (RRMS), 18 with primary progressive (PPMS) and ten with secondary progressive (SPMS). Forty-seven of the MS patients had been untreated for at least 30 days prior to sample collection. The remaining 12 were on various disease-modifying treatments at the time of sample collection, including interferon-beta, glatiramer acetate and dimethyl fumarate. For the subset of the 110 unique healthy volunteers with known demographic information, there were 61 males and 42 females; 57 whites, 35 African Americans, six Asians, one Hispanic, and one Native American. Ten healthy donors donated both saliva and blood, sixteen healthy donors donated blood, and the remainder of healthy donors we analyzed either PBMC or serum.

### Primers and probes

The NCBI reference genomes used to design the primer and probe sequences (Table 1) are NC_001664 for HHV-6A, strain U1102, and NC_000898 for HHV-6B, strain Z29. Ribonuclease P protein subunit P30 (*RPP30*) was used as a cellular housekeeping gene (Gene ID: 10556) [28]. The *RPP30* primers amplify a 62 base pair region, and the HHV-6 primers amplify an 89 base pair region *U57*, which encodes the major capsid protein. There is approximately 95–97% nucleotide homology between HHV-6A *U57* (Gene ID: 1487939) and HHV-6B *U57* (Gene ID: 1497059).

**Table 1:** Primer/probe sequences.

	Forward Primer (5′-3′)	Reverse Primer (5′-3′)	Probe (5′-3′)
*HHV-6A U57*	CCGTGGGATCGTCTAAAATTATAGATGT	CCACACTAGTCCGGACGGATAA	6FAM CTGGAACTGTATAATAGG MGBNFQ
*HHV-6B U57*	CCGTGGGATCGTCTAAAATTATAGATGT	CCACACTAGTCCGGACGGATAA	VIC CTGGAGCTGTACAACAG MGBNFQ
*RPP30*	GATTTGGACCTGCGAGCG	GCGGCTGTCTCCACAAGT	VIC CTGACCTGAAGGCTCT MGBNFQ

**Figure 1:**
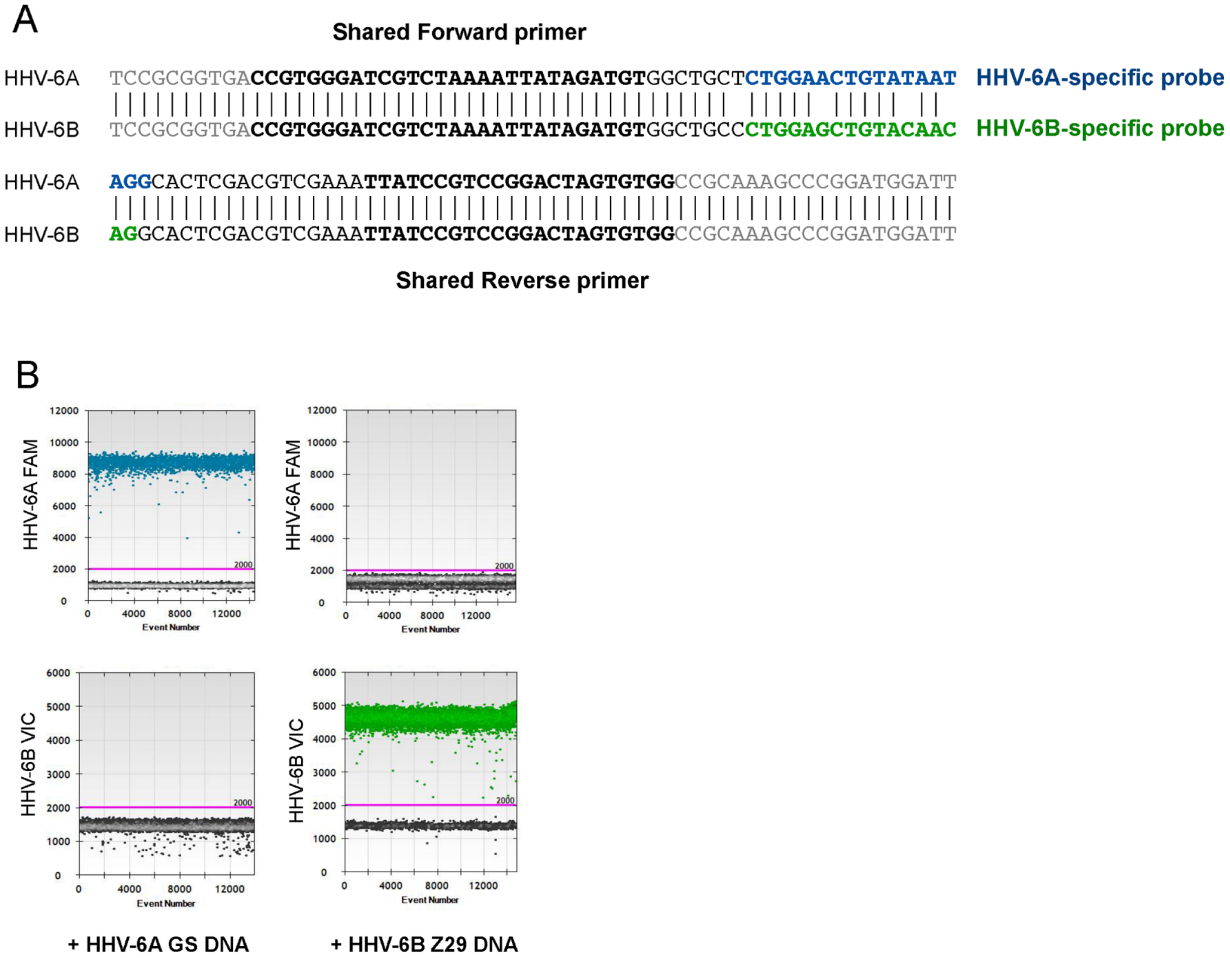
HHV-6A and HHV-6B duplex ddPCR assay design and specificity validation. (A) Primers were designed to amplify an 89 base pair region of *U57*, encoding the major capsid protein of HHV-6. The shared forward and reverse primers (in bold) amplify both HHV-6A and HHV-6B, while the probes are specific for each virus with a three base pair mismatch. The HHV-6A probe sequence is in blue, while the HHV-6B probe sequence is in green. (B) The probes distinguish HHV-6A and HHV-6B viral DNA with high specificity. The HHV-6A FAM-labeled probe binds HHV-6A DNA (blue droplets in left plot) but not HHV-6B DNA. Likewise, the HHV-6B VIC-labeled probe binds HHV-6B DNA (green droplets in right plot), but not HHV-6A DNA.

The primers amplify both HHV-6A and HHV-6B, while the differentially labeled fluorescent probes are specific for HHV-6A or HHV-6B. The HHV-6A probe is FAM-MGBNFQ-labeled, while the HHV-6B probe is VIC-MGBNFQ-labeled (see Figure 1). The housekeeping gene, RPP30, is VIC-MGBNFQ labeled. For each DNA sample, the HHV-6A and HHV-6B primers and probes were duplexed, while the *RPP30* primers and probe were separately singleplexed. When the primers and probes were duplexed (HHV-6A and HHV-6B) or singleplexed (*RPP30*), the final concentrations in each reaction were 900 nM per primer and 250 nM per probe. When the reaction was triplexed, the final concentrations of the HHV-6A and HHV-6B primers and probes remained as above, while the concentrations of *RPP30* were 450nM per primer and 125 nM for the probe.

### Sample collection/isolation

To isolate PBMC, whole blood was diluted with PBS, layered over a Ficoll gradient (Lympocyte Separation Medium, Lonza, Walkersville, MD) and spun at 12000 rpm for 30 minutes at room temperature (RT). The cells were washed, cryopreserved and stored in liquid nitrogen until DNA extraction. To isolate serum, whole blood was collected in a red top tube and spun at 2000 rpm for 10 minutes at RT; the serum was aliquoted and stored at –80°C until DNA extraction. Salivettes (Sarstedt, Newton NC) were used for saliva collection. Patients and healthy volunteers chewed on a cotton swab for two minutes, which was then placed in a capped plastic container and spun at 1100 rpm for 5 minutes. The saliva was stored at –20°C until DNA extraction.

### DNA extraction

DNA was extracted from saliva (200 μl) and PBMC or virus-infected cells (3×10^6^) using a DNeasy Blood and Tissue Kit (Qiagen, Valencia, CA) according to the manufacturer’s instructions. Buffer AVL was used for saliva extraction, while Buffer AL was used for PBMC extraction. DNA was extracted from serum (1 ml) using the QiaAmp Ultrasensitive Virus Kit (Qiagen, Valencia, CA) according to the manufacturer’s instructions. Elution volumes of purified DNA in Buffer AE were 150 μl for serum and saliva and 200 μl for PBMC.

### Viral Infections

HHV-6A (U1102) and HHV-6B (Z29) were propagated in the T-lymphoblastoid cell lines HSB-2 and SupT1, respectively, as previously described [33].

### Digital Droplet PCR

We used a modified version of the digital droplet PCR workflow described by Hindson, *et al.* [28]. Briefly, for each replicate, 5 μl DNA was digested with the restriction enzyme HindIII in NEB Buffer 2 (New England Biolabs, Ipswich, MA) for 30 minutes at 37°C, and then diluted 1:5 with molecular biology grade water. Dilution reduces salts in the digestion buffer that may interfere with PCR amplification. A 20 μl mixture of primers and probes, Bio-Rad 2X Supermix and the digested diluted DNA was emulsified with droplet generator oil (Bio-Rad, Hercules, CA) using a QX-100 droplet generator according to the manufacturer’s instructions. The droplets were then transferred to a 96 well reaction plate (Eppendorf, Hauppauge, NY) and heat-sealed with pierceable sealing foil sheets (Thermo Fisher Scientific, West Palm Beach, FL). PCR amplification was performed in this sealed 96 well plate using a GeneAmp 9700 thermocycler (Applied Biosystems, Grand Island, NY) with the following cycling parameters: 10 minutes at 95°C, 40 cycles consisting of a 30 second denaturation at 94°C and a 60 second extension at 59°C, followed by 10 minutes at 98°C and a hold at 12°C. Immediately following PCR amplification, droplets were analyzed using a QX100 droplet reader (Bio-Rad, Hercules, CA), in which droplets from each well are aspirated, streamed toward a detector and aligned for single-file two-color detection [28].

### DdPCR data analysis

Fluorescence data for each well were analyzed using QuantaSoft software, version 1.3.2.0 (Bio-Rad, Hercules, CA). Thresholds were determined manually for each experiment, according to the negative controls, which included a no template control and a negative sample. Droplet positivity was determined by fluorescence intensity; only droplets above a minimum amplitude threshold were counted as positive. Negative control DNA and a no template control were included in each experiment and resulted in zero positive droplets. For a given sample, target copies per μl were calculated by averaging over all replicate wells. Cellular DNA input was calculated by halving the number of *RPP30* copies, as there are two copies of *RPP30* per diploid cell. PBMC data are represented as viral copies per 10^6^ cells [34], while serum and saliva data are represented as viral copies per ml.

## Results

### Characterization of ddPCR for the detection of HHV-6A and HHV-6B

This assay was designed to multiplex HHV-6A and HHV-6B, such that coinfection within a given sample could be clearly visualized. The primers amplify the same region of *U57* from both viruses, while the fluorescently labeled probes distinguish between the two viruses by a three base pair mismatch (Figure 1A). This design minimizes differences in primer sensitivity and amplification kinetics that can lead to differences in the dynamic range of viral DNA quantitation [35]. To determine the optimal annealing temperature of these primers and probes, a temperature gradient experiment was performed using positive control DNA from HHV-6A and HHV-6B-infected cells. The optimal annealing temperature was determined to be 59°C (data not shown), which was used for all subsequent experiments. Primer/probe sequences are listed in Table 1.

Figure 1B shows several examples of one-dimensional (1D) ddPCR plots. The x-axis corresponds to the number of analyzed droplets (event number) and the y-axis corresponds to the fluorescence amplitude of the droplets. The purple line (amplitude 2000) is the manually set threshold, and droplets below this threshold are negative; that is, they do not contain amplifiable DNA. The maximum fluorescence amplitude of the probes differs for each fluorophore (FAM and VIC), and is further influenced by thermocycling conditions and the avidity of each probe for its target sequence.

The three base pair mismatch between the two probes confer a high level of specificity to each. Figure 1B depicts the results when HHV-6A DNA (HSB-2-infected cells, strain U1102) is tested with each probe. There is a positive droplet population (blue) in the left plot with the HHV-6A FAM probe, and the absence of positive droplets in the right plot with the HHV-6B VIC probe, indicating the specificity of the HHV-6A FAM probe for HHV-6A viral DNA. Likewise, when HHV-6B DNA (SupT1-infected cells, strain Z29) is tested with each probe, there is only a positive droplet population (green) in the right plot, with the HHV-6B VIC probe, indicating the specificity of the HHV-6B VIC probe for HHV-6B viral DNA.

### HHV-6A and HHV-6B coinfection detected in the peripheral blood and serum of healthy donors

**Figure 2:**
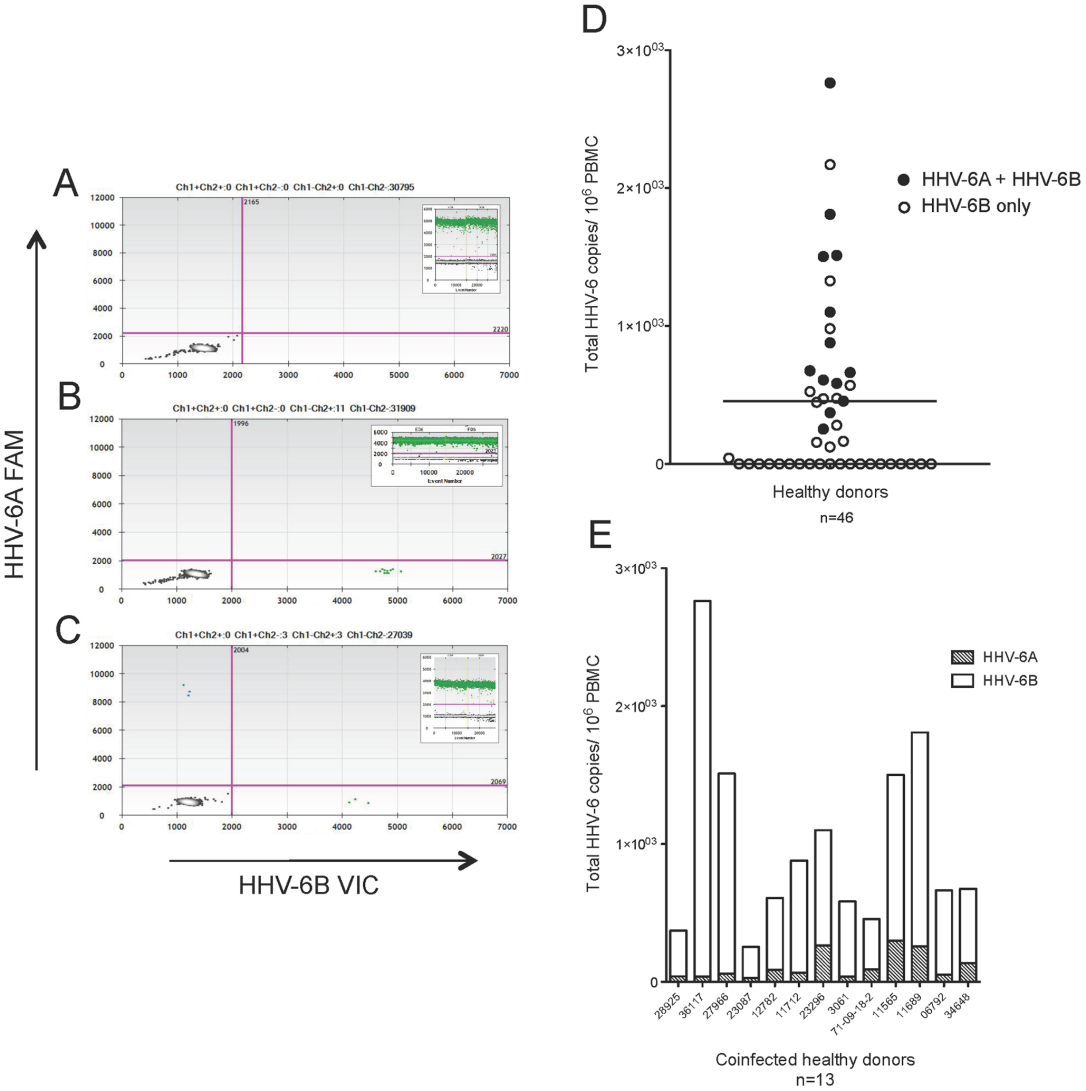
HHV-6 viruses detected in 57% healthy donor PBMC samples: 50% coinfection of HHV-6A and HHV-6B. Representative ddPCR plots with corresponding housekeeping gene (*RPP30*) as insets shown in A-C. (A) No positivity detected. (B) Only HHV-6B positivity (green droplets, lower right quadrant) detected. (C) Coinfection of HHV-6A and HHV-6B (blue droplets in upper left and green droplets in lower right quadrants, respectively) detected. (D) Group analysis of 46 healthy donor PBMC samples, with a mean (solid line) of 455 total HHV-6 copies/10^6^ cells. Each circle represents a donor. Closed circles represent detection of both HHV-6A and HHV-6B, while open circles represent detection of only HHV-6B. (E) The amount of HHV-6A and HHV-6B in the coinfected healthy donors (closed circles in D). The ratio of 6A/6B copies/10^6^ PBMC ranged from 0.01–0.25.

To extend previously published observations on the frequency of detection and quantitative range of viral DNA in healthy blood donors, 46 PBMC samples were analyzed by an HHV-6 multiplex ddPCR assay. Figures 2A-2C display representative two-dimensional (2D) plots, with HHV-6A FAM on the y-axis and HHV-6B VIC on the x-axis, and the corresponding 1D housekeeping gene (*RPP30* VIC) plots as insets. These data are representative of three outcomes: no HHV-6 positivity (Figure 2A), HHV-6 positivity with one virus (Figure 2B), or HHV-6 positivity with both viruses (Figure 2C). The presence of both HHV-6A and HHV-6B positive droplets is defined as coinfection. The 1D *RPP30* inset for all three donors displays a strongly positive droplet population, indicating the abundance of cellular material as expected from PBMC DNA. The *RPP30* concentration was used to calculate the number of viral copies per cell for each sample.

Figure 2D is a group analysis of the total HHV-6 viral copies per 10^6^ cells. Open circles represent the detection of only HHV-6B, while closed circles represent the detection of both viruses. The frequency of HHV-6 DNA detection in 46 healthy PBMC samples was 57% (26/46) with a range of 0-3,000 total HHV-6 copies per 10^6^ cells, consistent with other studies [20]. Of these 26 HHV-6 positive samples, HHV-6A and HHV-6B coinfection was detected in 13 (50%). The amounts of HHV-6A and HHV-6B within each coinfected sample are shown in Figure 2E. The 6A/6B ratio of copies/10^6^ PBMC ranged from 0.01–0.25, demonstrating that all healthy donor PBMC samples contained more HHV-6B compared to HHV-6A.

**Figure 3:**
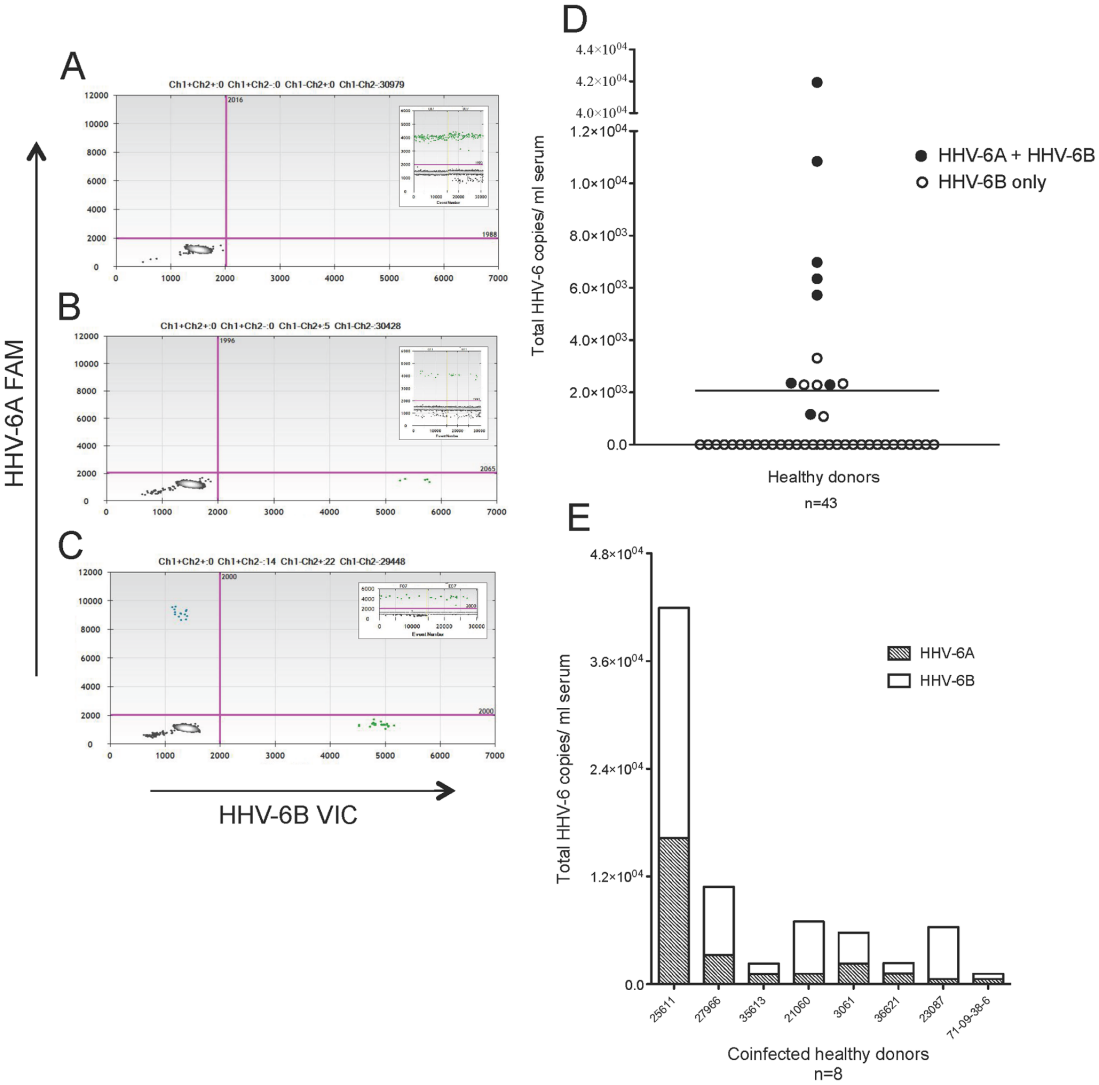
HHV-6 viruses detected in 30% healthy donor serum samples: 62% coinfection of HHV-6A and HHV-6B. Representative ddPCR plots with corresponding housekeeping gene (*RPP30*) as insets shown in A-C. (A) No positivity detected. (B) Only HHV-6B positivity (green droplets, lower right quadrant) detected. (C) Coinfection of HHV-6A and HHV-6B (blue droplets in upper left and green droplets in lower right quadrants, respectively) detected. (D) Group analysis of serum from 43 healthy donors, with a mean (solid line) of 2,069 total HHV-6 copies/ml. Each circle represents a donor. Open circles represent the detection of only HHV-6B, and closed circles represent the detection of both HHV-6A and HHV-6B. (E) The amount of HHV-6A and HHV-6B in the coinfected healthy donors (closed circles in D). The ratio of 6A/6B copies/ml serum ranged from 0.1 to 0.66.

Additionally, serum samples from 43 healthy blood donors were studied, and the results are displayed in Figure 3. Figures 3A-3C are representative 2D ddPCR plots, with the corresponding 1D *RPP30* plots as insets. The *RPP30* inset for all three donors displays a variably positive droplet population, with a reduced concentration compared to the PBMC DNA shown in Figure 2. Though *RPP30* was detected in most serum samples to varying extents, the data in Figure 3D are represented as total viral copies per ml. The frequency of viral DNA detection from 43 healthy serum samples was 30% (13/43) with a range of 0–42,000 copies/ml. These results are similar to a previous study that employed a highly sensitive PCR technique [36]. Of these 13 HHV-6 positive samples, HHV-6A and HHV-6B coinfection was detected in 8 (62%). The amounts of HHV-6A and HHV-6B within each coinfected sample are shown in Figure 3E. The 6A/6B ratio of copies/ml serum ranged from 0.1 to 0.66, demonstrating that all healthy donor serum samples contained more HHV-6B compared to HHV-6A. We did not observe correlations between HHV-6A and HHV-6B coinfection and gender or ethnicity (data not shown).

**Figure 4:**
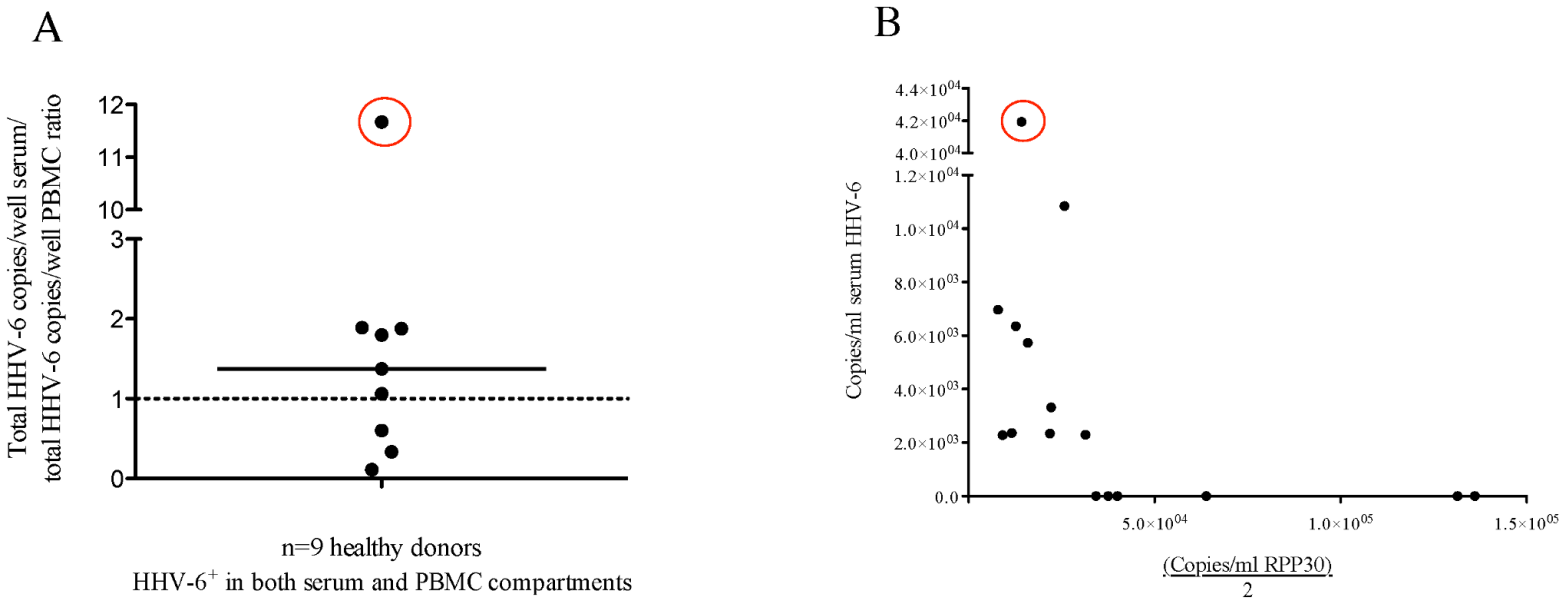
HHV-6 viral DNA detected in the serum may not reflect contamination from circulating blood cells. (A) Of sixteen normal donors with matched PBMC and serum samples, 11 were positive in at least one compartment and nine were positive in both compartments. For these nine donors, the data are displayed ratio of the copies per well of serum to the copies per well of PBMC. The dashed line at 1 represents an equal amount of virus in each compartment, while points above the line contain more virus in the serum compared to PBMC. The point circled in red is donor 25611, with the highest ratio of virus detected in the serum compared to PBMC. (B) In the serum samples of these sixteen donors, there is no correlation between copies of HHV-6 and copies of a cellular housekeeping gene *RPP30* (there are two copies of *RPP30* per cell). The point corresponding to donor 25611 is again circled in red, showing that despite a high serum/PBMC viral ratio, the serum did not contain increased cellular material relative to the other samples.

These PBMC and serum samples were from the same healthy donor in 26 cases- the results of 16 are shown in Figure 4, and the results of ten (who also donated saliva) are shown in Table 2. Of the 16 shown in Figure 4, 11 were positive in at least one compartment, and nine were positive in both. To examine the possibility of HHV-6 detection in the serum reflecting viral DNA released from circulating blood cells, we analyzed the ratio of viral copies in serum to viral copies in PBMC (these data are calculated before normalization to copies/cell or copies/ml). As shown in Figure 4A, eight of the nine samples cluster around a ratio of approximately one, suggesting approximately equal amounts of virus in the PBMC and serum. One normal donor (25611 circled in red) has a serum/PBMC ratio of approximately 11, demonstrating an abundance of viral DNA in the serum compared to the PBMC. In Figure 4B, the viral copies per ml of serum are plotted against the copies of cellular housekeeping gene, demonstrating that in the majority of samples, serum viral copies do not correlate with cellular housekeeping gene levels. These data support that viral DNA detected in the serum may not be cell associated (that is, independent of PBMC-associated virus).

**Table 2:** Multi compartment analysis of ten normal donors.

Healthy donor	PBMC (viral copies/10^6^ cells)	Serum (viral copies/ml)	Saliva (viral copies/ml)
68-26-8	ND a	ND	ND
10-09-1	473	ND	ND
09-02-7	ND	1,082	ND
10-05-4	ND	503	ND
09-38-6	ND	1,625 b	ND
70-64-9	320	ND	4,093
08-97-7	ND	ND	3,562
10-31-5	ND	ND	4,343
10-03-0	ND	ND	2,822
09-19-2	456 c	515	12,688

a ND  =  not detected.

b Coinfected: 580 copies/ml HHV-6A, 580 copies/ml HHV-6B.

c Coinfected: 92 copies/10^6^ cells HHV-6A, 369 copies/10^6^ cells HHV-6B.

### Identification of blood donors with probable chromosomally integrated HHV-6A or HHV-6B and applicability of ddPCR for screening

**Figure 5:**
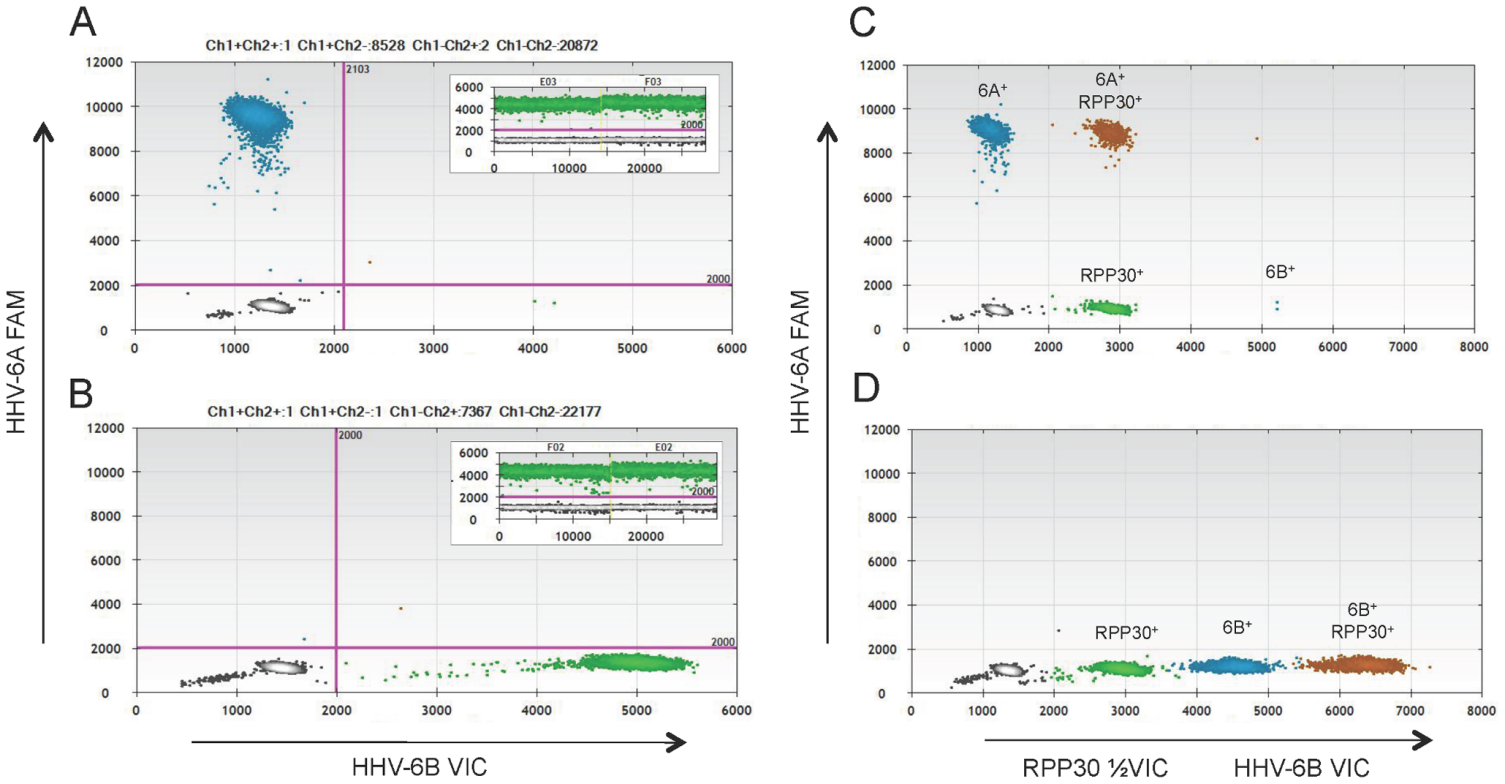
Identification of potentially chromosomally integrated blood donors using duplex and triplex ddPCR assays. (A) In a duplex reaction, donor 27867 was calculated to have 1.02 copies HHV-6A per cell. (B) In a duplex reaction, donor 28319 was calculated to have 0.98 copies HHV-6B per cell. The corresponding plots for the housekeeping gene *RPP30* (insets) were used to quantify the number of cells. (C) Triplex ddPCR of PBMC DNA from potentially chromosomally integrated donors. All three primer/probe sets (HHV-6A, HHV-6B and RPP30) can be assayed in a single well, using fluorescence intensities to distinguish between the droplet populations (labeled). In a triplex reaction, donor 27867 was calculated to have 1.09 copies HHV-6A per cell. (D) In a triplex reaction, donor 28319 was calculated to have 1.04 copies HHV-6B per cell.

In the assessment of healthy donor blood samples, we identified two individuals with unusually high levels of HHV-6 in their PBMC. Figure 5A displays PBMC DNA from donor 27867. Normalizing to the housekeeping gene *RPP30*, this individual was calculated to have approximately one copy of HHV-6A per cell (0.98 copies/cell). Figure 5B is a ddPCR plot of PBMC DNA from another donor, 28319. Normalizing to *RPP30*, this individual was calculated to have approximately one copy of HHV-6B per cell (1.02 copies/cell). This high concentration of HHV-6 suggests that these two individuals display a phenomenon known as chromosomally integrated (CI) HHV-6, which has been described in approximately 1% of the general population (reviewed in [37]).

The identification of these two individuals with probable CI HHV-6 led us to design a triplex reaction for HHV-6A, HHV-6B and *RPP30* (Figures 5C-5D). Triplexing enables a reduction in DNA input and increases system throughput, which are important for clinical settings in which CI HHV-6 may be significant. In this triplex condition, the concentrations of the *RPP30* VIC primers and probe were halved, allowing for the two VIC-labeled probes (HHV-6B and *RPP30*) to be distinguished by their fluorescence amplitudes. The calculated number of viral copies per cell was comparable whether the samples were duplexed (Figures 5A-5B) or triplexed (Figures 5C-5D). Donor 27867 was calculated to have 1.02 HHV-6A copies per cell when duplexed and 1.09 copies HHV-6A per cell when triplexed. Donor 28319 was calculated to have 0.98 copies HHV-6B per cell when duplexed and 1.04 copies HHV-6B per cell when triplexed.

### Elevated levels of HHV-6A and HHV-6B coinfection in the saliva of MS patients compared to healthy controls

**Figure 6:**
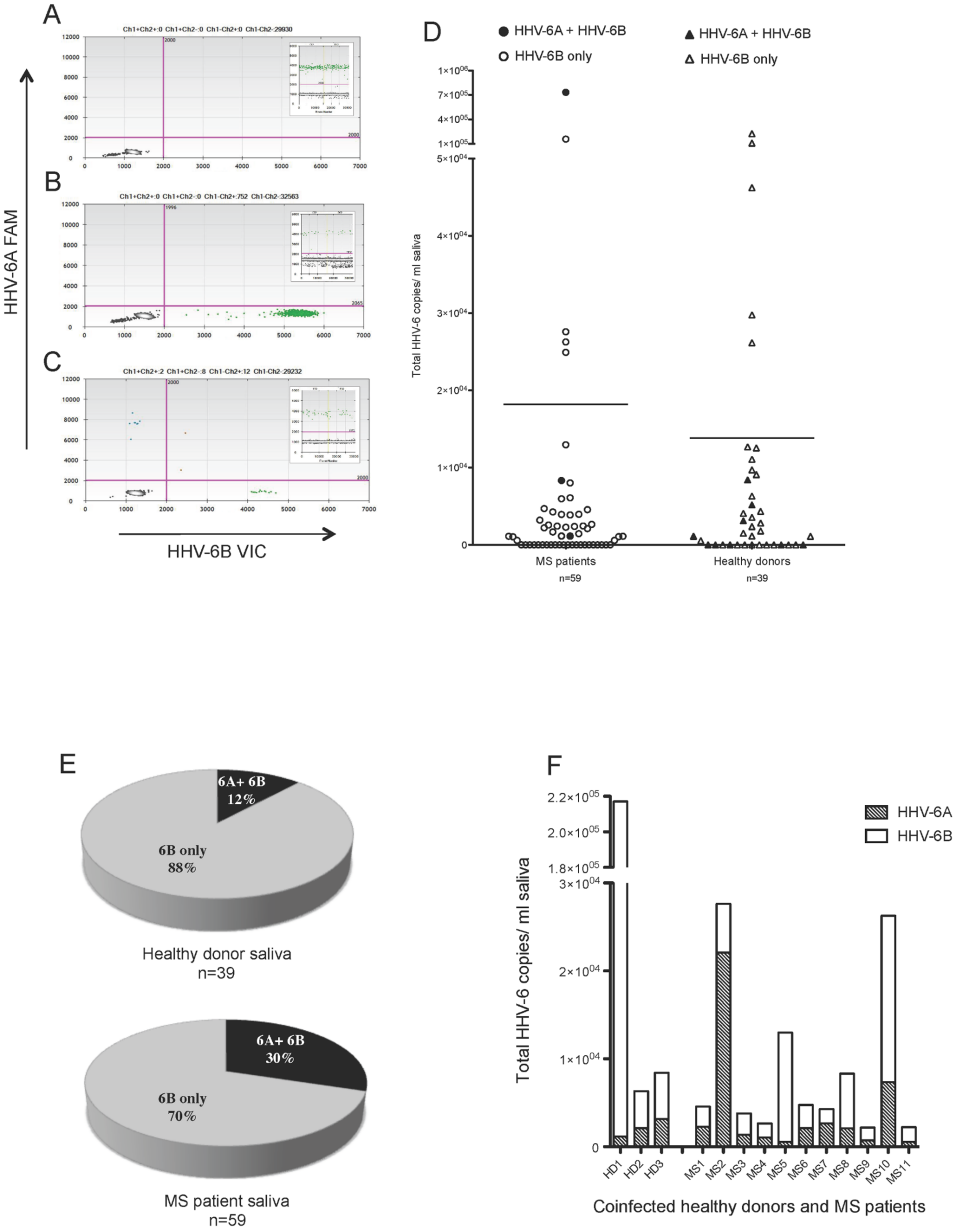
Increased HHV-6A and HHV-6B coinfection in MS patient saliva samples. Representative ddPCR plots with corresponding housekeeping gene (*RPP30*) as insets shown in A-C. (A) No positivity detected. (B) Only HHV-6B positivity (green droplets, lower right quadrant) detected. (C) Coinfection of HHV-6A and HHV-6B detected (blue droplets in upper left and green droplets in lower right quadrants, respectively). (D) Group analysis of total HHV-6 copies/ml in saliva of healthy donors (n = 39) and MS patients (n = 59). Lines represent the mean total viral copies: 1.38×10^4^ copies/ml for healthy donors and 1.82×10^4^ copies/ml for MS patients. Each symbol represents a donor. Open symbols represent the detection of only HHV-6B, and closed symbols represent the detection of both HHV-6A and HHV-6B. (E) The proportion of donors with HHV-6A and HHV-6B coinfection (dark shading) in saliva is significantly increased in MS patients compared to healthy donors (p = 0.04, Χ^2^ test). (F) The ratio of 6A/6B copies/ml saliva ranged from 0.005–0.6 in healthy donors and from 0.04 to 4.0 in MS patients.

HHV-6 viral DNA was also investigated in the saliva of healthy donors, as salivary glands are an important reservoir for HHV-6 [38], [39]. Figures 6A-6C are representative 2D ddPCR plots of three MS patient saliva samples. The *RPP30* insets for these three donors display variably positive green droplet populations compared to the PBMC DNA (Figure 2), but greater than what is observed for serum DNA (Figure 3). As with HHV-6 viral DNA in serum, the saliva data are expressed as viral copies/ml because similar to Cone *et al.* [20], in our study, salivary cellular DNA levels did not correlate with viral DNA levels (data not shown). Collectively, these results demonstrate HHV-6 in the saliva of 67% (26/39) healthy donors. Of these HHV-6 positive saliva samples, only 12% (3/26) were found to be coinfected with HHV-6A and HHV- 6B (summarized in Table 3), lower than what was observed in the PBMC (Figure 2) or serum (Figure 3). We did not observe correlations between coinfection and gender or ethnicity (data not shown). This high frequency of detection [19], [21], [40], [41] and range of viral copies/ml saliva [20] are both consistent with other published reports.

**Table:** **Table 3.** Summary of HHV-6 detection by ddPCR HHV-6 positive samples.

		HHV-6 positive/Total samples (%)	Total viral copies Mean (Range)	HHV-6B only (%)	HHV-6A + HHV-6B coinfection (%)
**PBMC**	Healthy donors	26/46 (57)	455 copies/10^6^ cells (0 – 3×10^3^)	13/26 (50)	13/26 (50)
**Serum**	Healthy donors	13/43 (30)	2.07×10^3^ copies/ml (0 – 4.2×10^4^)	5/13 (38)	8/13 (62)
**Saliva**	Healthy donors	26/39 (67)	1.38×10^4^ copies/ml (0 – 2.3×10^5^)	23/26 (88)^a^	3/26 (12)^a^
	MS patients	37/59 (63)	1.82×10^4^ copies/ml (0 – 7.3×10^5^)	26/37 (70)^a^	11/37 (30)^a^

a p = 0.04 (χ^2^).

Of the HHV-6 viruses, HHV-6A has been suggested to be more neurotropic than HHV-6B [33], [42] and is associated with MS [43], [44]. Therefore, we asked if the level of coinfection (which in this study is defined as the presence of HHV-6A in addition to HHV-6B) is increased among MS patients compared to healthy donors (Figure 6D). The rates of viral detection in saliva were similar between MS patients (63%) and healthy donors (67%), with a mean total viral load of 1.82×10^4^ copies/ml for MS patients and 1.38×10^4^ copies/ml for healthy donors (summarized in Table 3). However, there was a significantly elevated prevalence of coinfection in MS patient saliva (30% (11/37)) compared to the healthy controls (12% (3/26)) (Χ^2^ test, p = 0.04) (Figure 6E), suggesting that the difference between MS patients and controls may be the increased presence of HHV-6A in addition to HHV-6B. The amounts of HHV-6A and HHV-6B within each coinfected sample are shown in Figure 6F. The 6A/6B ratio of copies/ml saliva ranged from 0.005-0.6 in healthy donors and from 0.04 to 4.0 in MS patients, demonstrating that in healthy donors, all coinfected samples contained more HHV-6B compared to HHV-6A, while in MS patients, a subset of the coinfected samples contained more HHV-6A than HHV-6B (2/11). In this particular MS cohort, there was no correlation between coinfection and expanded disability status scale (EDSS) score, brain lesion load or contrast enhancing lesions around the time of saliva collection (data not shown).

## Discussion

The HHV-6 viruses are detected at low levels in the majority of healthy individuals. This is supported by the widespread—but controversial—use of nested PCR to establish the frequency of HHV-6 in the blood of healthy donors [19], as well as data demonstrating that viral detection in blood increases with increasing cellular input [19], [20], [24]. Such low levels of virus render the detection of viral DNA highly subject to both the sample integrity and assay sensitivity, and may underlie discrepancies in the literature regarding the frequency of HHV-6 detection among healthy individuals.

In this paper, we report a novel HHV-6 detection method using a third generation quantitative PCR technology, ddPCR. DdPCR is ideal for detecting low frequency events, such as HHV-6 viral DNA in healthy donor samples, because it partitions a sample into discrete droplets, thereby minimizing random sampling error. Moreover, increasing the number of replicates proportionally increases the quantitative dynamic range; this ability to extend the lower limits of detection is ideal for assessing low-level targets [28], and important for detecting two viral species that may be present in different quantities.

We detected HHV-6 viral DNA in the PBMC of 57% (26/46) of healthy donors, consistent with a number of previous studies [20], [45]. When considering prevalence data in a cross-sectional study such as this one, it is important to note that detectable viral DNA levels within an individual may fluctuate over time. This was demonstrated in a longitudinal study of healthy children, which reported that over several years, viral DNA was detected intermittently in the PBMC of 76% children, but consistently detected in the PBMC of only 12% of children [46].

In our study of healthy donor PBMC, we fortuitously identified two individuals with probable CI HHV-6, one with HHV-6A and the other with HHV-6B, though analysis by fluorescent *in situ* hybridization (FISH) is necessary to confirm chromosomal integration. The precise mechanisms and clinical implications of chromosomally integrated HHV-6A and HHV-6B are active areas of study [47], [48]. From studies of healthy blood donors in the US and UK, the incidence of HHV-6 chromosomal integration in the general population is thought to be approximately 1% [37]. We believe that the percentage of potentially chromosomally integrated individuals as calculated from this study (2/48, or 4%) reflects low numbers of surveyed PBMC.

In the healthy blood donor with probable integrated HHV-6A (1.02×10^6^ copies HHV-6A per 10^6^ cells) we also detected HHV-6B viral DNA (306 copies HHV-6B per 10^6^ cells). These data are consistent with a recent report of HHV-6B detection in two individuals determined to be CI HHV-6A [35]. Notably, we detected these HHV-6B positive droplets both when the sample was duplexed (306 copies HHV-6B/10^6^ cells) and triplexed (401 copies HHV-6B/10^6^ cells). These data highlight the suitability of ddPCR for assessing coinfection, as low copy numbers of one viral species can be detected and reproducibly quantified in the presence of excess copies of the other viral species.

The detection of viral nucleic acid in blood may reflect active viral replication, chronic persistence or latency [1]. We detected HHV-6 DNA in 30% (13/43) serum samples from healthy adult donors. These results are higher than many previous studies [21], [49]–[52] that employed conventional PCR approaches and reported undetectable levels of HHV-6 DNA in healthy donor serum. However, a frequency of 30% agrees with several other studies, including one that used a highly sensitive PCR ELISA assay and reported HHV-6 DNA in the serum of 21% (5/24) healthy Canadian donors [36] and another that reported 20% detection among healthy Jordanian controls [53]. It is currently thought that HHV-6 DNA detected in the serum indicates an active infection, but recent work cautions that viral DNA in the serum may in fact reflect DNA released from circulating blood cells [54]. While cellular material in serum can indicate the presence of genomic DNA contaminants [55], we found no correlation between viral copies/ml serum and the cellular housekeeping gene (a subset shown in Figure 4B), suggesting that viral DNA we detected in serum may not be cell-associated. An active infection can also be hypothesized based on the ratio of virus in the serum to virus in circulating peripheral blood cells (Figure 4A). For example, most individuals in our study had a ratio of approximately one, but one individual had a ratio of 11. This 11-fold increase of virus in the serum compared to PBMC supports further studies on this individual to determine whether there is active or infectious virus in this compartment.

A major finding of this report is the high frequency of HHV-6A and HHV-6B coinfection in healthy adult donors. There are few published studies on coinfection of the HHV-6 viruses, likely because many early reports did not distinguish between HHV-6A and HHV-6B, though the literature is shifting towards this distinction [56]. Even in studies that report coinfection [21], [57], there may be an underestimation of the frequency. For example, in one study of 44 adult PBMC samples, coinfection was detected in 18% of the HHV-6 positive samples [57]. This is comparatively lower than our data, as we detected coinfection in 50% (13/26) of HHV-6 positive PBMC samples. We also report coinfection in 62% (8/13) of HHV-6 positive serum samples, and are not aware of other studies that observed this in healthy donor serum. This high percentage likely reflects the low denominator, and should be interpreted with caution.

In addition to PBMC and serum, we also analyzed the HHV-6A and HHV-6B viral loads in saliva. The salivary glands are a known reservoir for HHV-6 [38] and saliva is an easily collected clinical sample. Among healthy donors, we observed the lowest incidence of HHV-6A and HHV-6B coinfection in this compartment (12%, relative to 50% in PBMC and 62% in serum). However, this observation may be population-specific, as a recent study of HHV-6 in the saliva of healthy Brazilian donors reported twice the frequency of HHV-6A compared to HHV-6B [25].

As we observed higher viral loads in the saliva compared to the blood of healthy donors, we chose this sample type to compare HHV-6 levels between healthy donors and patients with MS, a neurologic disease associated with HHV-6 (reviewed in [58]). Despite comparable detection frequencies and total viral loads between healthy donor and MS saliva, we detected significantly increased HHV-6A and HHV-6B coinfection in the MS samples. These data extend observations from a previous study in which we detected coinfection in a subset of HHV-6 positive saliva samples from MS patients (6%), but not healthy controls [21]. The observation of increased detection of HHV-6A in MS patients is also consistent with other studies suggesting that this viral species is more prevalent among MS patients compared to healthy donors [21], [59].

In this report, coinfection is defined by the presence of HHV-6A in addition to HHV-6B, as singly infected samples were determined to be HHV-6B. This may reflect that HHV-6B is the predominant species of childhood infection among our donor population. The implications of coinfection should be considered in light of clinical reports suggesting that the abundance of detectable HHV-6A may be pathophysiologic. For example, in a study of renal transplant patients [60], the frequency of HHV-6A was significantly higher in patients with active infections, while the frequency of HHV-6B was significantly higher in patients and healthy donors with latent infections. In a separate study of critically ill patients, 54 of 101 had detectable HHV-6 viral DNA, and 53 of the 54 were positive for HHV-6A. The authors suggest that the selective reactivation of HHV-6A reflects the dominance of this viral species under illness-related stress [61].

As HHV-6A may be more active than HHV-6B [52], [60] and studies demonstrate different susceptibilities of each virus to therapeutics [4], quantitating the extent of coinfection may prove clinically relevant. It may be insufficient to assess only the presence or absence of each species in a given sample, as we determined great variability in the viral copy ratio of HHV-6A to HHV-6B among our coinfected healthy and MS saliva samples (0.005 to 4.0). It remains to be seen in a larger study whether there are clinical correlates to this ratio.

In summary, we aimed to address the frequency of HHV-6A and HHV-6B coinfection by designing a multiplex digital droplet PCR assay to comparably amplify both viruses. We investigated blood and saliva samples from healthy adult donors and adult MS patients and demonstrate a heretofore-underappreciated high frequency of coinfection of these two viruses. Larger studies are required to learn to what extent the variability of frequency positive and coinfection are related to the site (PBMC, serum or saliva) versus the population, though our cohort in Table 2 (ten donors from which we collected PBMC, serum and saliva) suggests that viral positivity is related to the site as opposed to the population. Moreover, larger studies are required to determine if there are clinical correlates of HHV-6A and HHV-6B coinfection (for example, markers of inflammation) and if the frequency of either virus is affected by treatment. As we understand more about each virus in health and disease, this observation of coinfection may have profound implications.
